# Age differences in alcohol and music consumption among Japanese nonproblem drinkers

**DOI:** 10.1038/s41598-025-26809-0

**Published:** 2025-12-07

**Authors:** Naoki Kato, Shunji Oshima, Katsumi Watanabe

**Affiliations:** 1 Core Technology Laboratories, Asahi Quality & Innovations, Ltd., 1-21 Midori 1-chome, Moriya, Ibaraki 302-0106 Japan; 2 Sustainable Technology Laboratories, Asahi Quality & Innovations, Ltd., 1-21 Midori 1-chome, Moriya, Ibaraki 302-0106 Japan; 3 Faculty of Science and Engineering, Waseda University, 3-4-1 Okubo, Shinjuku-ku, Tokyo, 169-8555 Japan

**Keywords:** Alcohol consumption, Music consumption, Cognitive emotion regulation strategy, Psychosocial development, Psychology, Human behaviour

## Abstract

Alcohol use and music use were examined in relation to emotion regulation; however, most studies examined these two factors separately. We examined how people of different ages drink alcohol and listen to music within the context of emotion regulation. The following survey data was analyzed among Japanese nonproblem drinkers (20–59 years, *N* = 1279): How often people drink alcohol and listen to music, Tendencies to use them when experiencing negative emotions, Drinking Motives Questionnaire-Revised, Brief Music in Mood Regulation Scale, Cognitive Emotion Regulation Questionnaire, Ten-Item Personality Inventory, and Perceived Stress Scale. The results showed that younger adults tended to drink less than older adults. They listened to music more often when they were feeling negative emotions. Mediation analysis suggested younger adult’s lower extraversion and lower positive reappraisal strategies were associated with higher stress levels, leading them to use music for diversion. Furthermore, people who consume alcohol to regulate emotions showed higher tendency to listen to music within the context of emotion regulation, which was the case for both younger and older participants. This study helps us understand how people of different ages use different methods to manage their emotions when faced with changes in their social and emotional environments.

**Supplementary Information:**

The online version contains supplementary material available at 10.1038/s41598-025-26809-0.

## Introduction

Alcohol and music have co-existed since ancient times^1^. Alcohol is sold at modern live music venues and typically consumed while music is being played at traditional festivals. Apart from social places, such as festivals or live music venues, alcohol and music can be accessed from familiar places (e.g., alcohol can be bought at supermarkets, while one can listen to music on personal smartphones using streaming services). However, one of the key differences between alcohol and music consumption is many countries impose a legal drinking age. Although legal drinking age differs, it is set to 20 in Japan, where this study was conducted.

Regarding the relationship between drinking behavior and age, scholars in Japan have reported differences in alcohol consumption rates between age groups, and data from 2019 have demonstrated that the drinking rates for individuals in middle adulthood (40–59 years) are higher than for those in young adulthood (20–39 years)^2^.This trend in differences between age groups was generally similar to that observed in 1989^2^, which represents approximately one generation earlier (30 years prior to 2019). Furthermore, previous research for Japanese has examined not only age differences in drinking behavior (drink/not drink) but also age differences in why people drink. For example, Imada et al.^3^ developed the Drinking Motive Questionnaire for Japanese (DMQ-J) for people aged 20–69 years and reported that social motives (e.g., drinking to enhance interpersonal relationships) decrease with age. Other scales have also been developed to measure the drinking motives of Japanese people. In particular, Okada^4^ formulated the Japanese version of the widely used Drinking Motives Questionnaire-Revised (DMQ-R), which was developed by Cooper^5^. The DMQ-R was originally intended for adolescents, but later research reported its applicability to older adults^6^. This scale consists of four subscales (enhancement, social, coping, and conformity) derived from a two-dimensional framework (valence: positive or negative; source: internal or external)^7^. Okada^4^ reported that the subscale score for enhancement was significantly higher than that for social among Japanese adults in middle adulthood (40 s and 50 s). This finding was consistent with Imada et al.^3^.

Beyond age differences, reports indicate that drinking motives also vary across cultures. Mackinnon et al.^8^ conducted a large-scale study across 10 countries using a shortened version of the DMQ-R and, based on prior cross-cultural frameworks (e.g., Hofstede et al.^9^), grouped these countries into relatively individualistic (Switzerland, Hungary, Canada, the Netherlands, the UK and Ireland, and the USA) and relatively collectivistic (Portugal, Mexico, Brazil, and Spain) categories. They found that individuals from the relatively individualistic countries were more likely to drink for social and enhancement motives than those from the relatively collectivistic countries (this distinction is relative within the set of countries examined and does not imply an absolute global ranking). However, this study did not include East Asian countries such as Japan, and the participants were limited to undergraduates. Therefore, measuring drinking motives among East Asian individuals such as Japanese using the DMQ-R, including a broader age range, is meaningful for accumulating insights into both age difference and cultural difference in drinking motives.

In Japan, workplace drinking events (‘nomikai’) are deeply embedded in corporate culture and serve not only social but also emotional regulatory functions, particularly for older adults. These events are seen as opportunities to release emotional tension within hierarchical relationships^10^. On the other hand, karaoke—widely popular among younger and older generations alike—provides a culturally normative avenue for expressing and modulating emotional states^11^. Such practices highlight the need to consider culturally embedded emotion regulation practices when examining age differences in alcohol use and music use.

Turning to music, the literature highlights the relationship between music and age. For example, Chamorro-Premuzic et al.^12^ conducted a study on individuals aged 18–64 years, mainly European/Caucasian, and reported that music consumption was negatively correlated with age. In addition to general music consumption, the emotional use of music as defined by the Uses of Music Inventory^13^ was negatively correlated with age^12^. Mood, like emotion, refers to a human affective state, but there are differences between the two concepts: emotion typically refers to short-duration, event-related affective states that involve physiological and behavioral responses, while mood represents a more enduring affective condition that is not necessarily tied to a specific event^14^. However, in studies that examine the effects of music on emotion regulation, emotion includes a range of terms, from feeling to mood^15^. Saarikallio^16^ developed the Music in Mood Regulation (MMR) scale to measure mood regulation through music among Finnish adolescents aged 10–20 years. The author later formulated a brief version of the same scale (B-MMR)^17^. The B-MMR consists of seven subscales (entertainment, revival, strong sensation, diversion, discharge, mental work, and solace) and has been translated into Italian and Japanese^18,19^. Scholars proposed that the B-MMR can be expanded beyond adolescence and reported a significant positive correlation between age and the subscale score for strong sensation, which refers to using music to seek intense emotional experiences^18^. In addition, previous studies pointed to differences in age in general cognitive emotion regulation strategies. For example, Bailly et al.^20^ emphasized that the older the age group, the lower the score on the rumination subscale of the Cognitive Emotion Regulation Questionnaire (CERQ)^21,22^, which measures one’s cognition when experiencing negative or unpleasant events. These results demonstrate the complex relationship among age, music consumption, and emotion regulation.

As noted earlier, the consumption of alcohol/music and their underlying motives/strategies are related to age. However, studies tend to examine age differences in their use separately, especially in the context of emotion regulation. Consequently, it remains unclear whether individuals who use alcohol to regulate emotions also tend to use music for the same purpose, and whether age moderates this within-person association. Clarifying this issue may advance theory by revealing whether alcohol and music—characterized by different sensory modalities—function as alternative or complementary means of emotion regulation, and by indicating whether sensory preferences in emotion regulation shift with age. Such insights would refine our understanding of age-related patterns in the selection and combination of emotion regulation strategies. In addition, despite the reported effectiveness of early interventions on problem drinking (e.g., Refs.^23,24^), to our knowledge, no studies have been conducted to comprehensively examine the engagement of nonproblem drinkers with alcohol and music when experiencing negative emotions.

To address this research gap, the current study aimed to comprehensively explore age differences in the consumption of alcohol/music, especially in the context of emotion regulation, with a focus on Japanese nonproblem drinkers. Specifically, data on the general frequency of alcohol/music consumption, tendency to use alcohol/music when experiencing negative emotions, the DMQ-R, the B-MMR, and the CERQ were obtained by conducting an online survey on individuals aged 20–59 years. Data on personality and stress levels were also obtained and analyzed to interpret the results. This is because, in addition to the relationship between personality and stress levels^25^, other studies have reported relationships between alcohol use and personality^26^ and between music use and personality^13^. Correlation analysis and mediation analysis were conducted between age and above items. Considering previous studies showing that younger individuals drink less alcohol and listen to more music, it is plausible that individuals may increasingly rely on alcohol rather than music for emotion regulation as they age, if alcohol and music serve as alternative rather than complementary means of emotion regulation.

## Materials and methods

### Participants and procedure

The researchers conducted an online survey with funding from Asahi Quality & Innovations, Ltd (AQI). The participants were recruited from the survey panels of Cross Marketing Group Inc. (a major Internet survey company in Japan). The recruitment period spanned from July 9, 2024, to July 31, 2024.

The participants were first briefed about the objective and procedure of the study, and informed consent was obtained online. They then completed the demographic survey, which sought the following information: gender, age, marriage, cohabitants/cohabiting families, occupation, and occupation of family members. Participants whose occupations, or those of their family members, fell within the food and beverage, food service, mass media, or marketing research industries were deemed ineligible and were excluded from the study. These exclusions were predefined to reduce the risk that occupational expertise or industry attitudes might introduce systematic bias in responses when completing the questionnaire. The sample size for the survey was determined based on AQI’s budget, and a total of 1530 participants aged between 20 and 59 years responded to the questionnaires described in “Measures” (*M*_*age*_ = 40.55, *SD* = 9.96; female: 56.4%; age range/distribution: 20s: 15.9%, 30s: 33.6%, 40s: 28.1%, and 50s: 22.4%). The age range of participants was determined according to the legal drinking age and Okada’s study^4^.

Specifically, the data analysis was limited to respondents with AUDIT scores below 8 (see “Alcohol use disorders identification test” for details), because the study focused on age differences in how nonproblem drinkers use alcohol and music—either separately or in combination—when experiencing negative emotions, and their interrelationships. As a result, the number of participants decreased to 1,279 (*M*_*age*_ = 40.20, *SD* = 9.94; female: 59.81%; age range/distribution: 20s: 16.18%, 30s: 34.79%, 40s: 27.29%, and 50s: 21.74%; Supplementary Table S1).

Participants who responded to all items were given an Amazon gift certificate worth 2,000 yen as compensation. The Ethics Committee of User Life Science, Ltd. approved the study protocol (reference number: ULS 202406).

### Measures

#### Alcohol use disorders identification test

AUDIT is a screening tool for alcohol use disorders developed by the WHO; Hiro^27^ developed its Japanese version. AUDIT consists of 10 items and produces a full score of 40 points. The participants completed the AUDIT, and those who met the criteria for alcohol use disorders were excluded from the main analysis. The criterion for alcohol use disorder was set to a total score of 8 or more points, which is the standard as per the AUDIT guidelines^28^.

#### Self-perceived ability to metabolize alcohol

To measure participants’ self-perceived ability to metabolize alcohol (commonly understood in Japan as being “strong” or “weak” in drinking), the participants provided the most applicable responses to the following questions: “In terms of your perceived ability to handle alcohol, please select the response that closely matches your perception” (1 = *Very weak*, 5 = *Very strong*). Participants who never consumed alcoholic beverages were provided with a separate option: “Never had a drink.” Out of the 1279 participants, 56 never consumed alcoholic beverages.

#### Frequency of drinking alcoholic beverages

Drinking frequency can also be measured by AUDIT item 1; however, this item uses a five‑category ordinal frequency scale following WHO materials^28^. Because a continuous measure of drinking frequency was required for correlation analyses, we collected a separate variable, distinct from AUDIT item 1, for this purpose. The participants self-reported the number of days that they drank alcoholic beverages in the past 30 days. This item was answered in response to the following questions: “How often do you usually drink alcoholic beverages? Please answer this question by totaling all of these contexts, including who you drink with and where you drink and so on.” and “How often do you usually drink alcoholic beverages alone?” Data analysis considered not only these two frequencies but also differences between them.

#### Frequency of listening to music

The participants self-reported the number of days that they listened to music in the past 30 days. This item was answered in response to the following question: “How often do you usually listen to music of your own volition? Please answer without including background music that plays in shops, on TV, online videos, etc., regardless of your own intentions.”

#### Tendency of drinking alcoholic beverages/listening to music when experiencing negative emotions

Participants self-reported the tendency of conducting the following behaviors when experiencing negative emotions: drinking alcoholic beverages without listening to music, drinking alcoholic beverages while listening to music, and listening to music without drinking alcoholic/caffeinated beverages. Although it was outside the scope of the study, the reason for asking about this tendency through the description “listening to music without drinking alcoholic/caffeinated beverages” was that the participants were also asked about the contents of their caffeinated beverages in the online survey. These tendencies were answered in response to the following questions:

When you are experiencing negative emotions, do you sometimes do the following things? In terms of listening to music, this includes watching music videos and excludes background music played in shops and other places without your consent. When you are doing these things, you may or may not be doing other things at the same time (e.g., eating and talking to someone). You are encouraged to respond spontaneously.

Unlike other measures, we were unfortunately unable to find comparable measures for alcohol use, alcohol use in combination with music, and music use alone when experiencing negative emotions. Therefore, these variables were measured using the above instructions which is referred to the CERQ-short-RJ (see “Revised Japanese version of the cognitive emotion regulation questionnaire (CERQ-short-RJ)” for details) and a five-point Likert scale (1 = *Never do it*, 5 = *Always do it*).

#### Japanese version of the drinking motives questionnaire-revised (DMQ-RJ)

To measure potential motives for drinking alcoholic beverages, the participants completed the Japanese version of the DMQ-R (DMQ-RJ; Cooper^5^; Okada^4^). This scale consists of 20 items and comprises four subscales, namely: *enhancement*, which is internally focused and directed toward oneself, pursuing positive incentives; *social*, which is externally focused and directed toward socially significant others, pursuing positive incentives; *coping*, which is internally focused and directed toward oneself, avoiding negative incentives; and *conformity*, which is externally focused and directed toward socially significant others, avoiding negative incentives^7^. Items were rated using a five-point Likert scale (0 = *Almost Never*/*Never*, 4 = *Almost always*/*Always*). The participants who do not typically drink alcoholic beverages at all were instructed to select 0 = *Almost Never*/*Never*.

#### Japanese version of the brief music in mood regulation scale (B-MMR-J)

To measure the extent to which the participants resorted to the specific strategies for mood regulation through music, they completed the Japanese version of the B-MMR (B-MMR-J; Saarikallio^16,17^; Shoda et al.^19^). This scale consists of 21 items and comprises seven subscales, namely: *entertainment*, which denotes the use of music to create a pleasant atmosphere and evoke feelings of happiness, thereby maintaining or enhancing an existing positive mood; *revival*, which refers to listening to music for the purpose of relaxation and restoration of energy when experiencing stress or fatigue; *strong sensation*, which involves engaging with music to elicit intense emotional experiences; *diversion*, which is characterized by the use of pleasant music to distract from and alleviate unwanted thoughts and feelings; *discharge*, which pertains to the release of anger or sadness through music that conveys these emotions; *mental work*, which signifies employing music as a framework for mental contemplation and reappraisal of emotional preoccupations; and *solace*, which represents listening to music to foster a sense of acceptance and understanding during periods of sadness or trouble^17^. Items were rated using a five-point Likert scale (1 = *Strongly Disagree*, 5 = *Strongly Agree*).

#### Revised Japanese version of the cognitive emotion regulation questionnaire (CERQ-short-RJ)

To measure cognitive emotion regulation strategies, specifically, one’s cognition when experiencing negative or unpleasant events, the participants completed the CERQ-short-RJ^29^; see CERQ^21^, and CERQ-short^22,30^. This scale consists of 18 items organized into nine subscales as follows: *self-blame*, which refers to thoughts of blaming oneself for an event; *other-blame*, which refers to thoughts of putting the blame for an event on others; *rumination*, which involves thinking about feelings and thoughts associated with negative events; *catastrophizing*, which refers to thoughts of explicitly emphasizing the terror of an experience; *refocus on planning*, which involves thinking of the next steps to take and how to address a negative event; *positive reappraisal*, which refers to thoughts of attaching a positive meaning to an event in terms of personal growth; *putting into perspective*, which involves thoughts of playing down the severity of an event or emphasizing its relative importance compared with other events; *acceptance*, which refers to thoughts of accepting the occurrence of an event and resigning oneself to what has happened; and *positive refocusing*, which involves thinking about joyful and pleasant issues instead of focusing on an actual negative event. Items were rated using a five-point Likert scale (1 = *(almost) never*, 5 = *(almost) always*).

#### Japanese version of the ten item personality inventory (TIPI-J)

To measure the Big Five personality traits, the participants completed the Japanese version of the Ten-Item Personality Inventory (TIPI-J^31,32^). The scale consists of 10 items and five subscales, namely, neuroticism, extraversion, openness, agreeableness, and conscientiousness. Items were rated using a seven-point Likert scale (1 = *Disagree strongly*, 7 = *Agree strongly*).

#### Japanese version of the perceived stress scale (PSS-J)

To measure general stress, the participants completed the 10 items of the Japanese version of the Perceived Stress Scale (PSS-J^33,34^). Items were rated using a five-point Likert scale (0 = *Never*, 4 = *Very often*), and the total score was used for data analysis.

### Statistical Analysis

Data were analyzed using IBM SPSS Statistics version 29.0.1. The study considered the large sample size and set the significance level to 1% when conducting statistical hypothesis testing. It then examined and interpreted effect size in addition to *p*-value. The interpretation of the absolute value of the effect size followed the classification of Mizumoto and Takeuchi^35^, using |*r*|≥ 0.10 for correlations and |*d*|≥ 0.20 for mean differences as indicative benchmarks rather than strict cut-offs, as these thresholds were suggested by Mizumoto and Takeuchi as reference values. These thresholds apply only to bivariate correlations and group differences; they were not applied to β or indirect effects in mediation analyses, where significance was determined by whether bias-corrected and accelerated (BCa) bootstrap 95% CIs excluded zero.

To describe screening outcomes prior to applying the AUDIT < 8 inclusion criterion, we compared the prevalence of AUDIT risk categories (≥ 8 vs. < 8) by gender and age range using the Chi-square test (Supplementary Table S1). All analyses thereafter used the nonproblem drinker sample (AUDIT < 8). Gender differences in the items/subscale scores described in “Measures” were assessed using Welch’s *t*-test (Supplementary Table S2). Meanwhile, we computed Z-scores for each scale separately: the DMQ-RJ and B-MMR-J. Specifically, for each participant, item scores within each scale were standardized (within-person Z-scores) using that participant’s own mean and standard deviation across all items of that scale. This approach allows interpretation of subscale scores relative to the participant’s overall response pattern within the same scale, minimizing the influence of absolute alcohol or music consumption frequency. These Z- scores were then used in subsequent analyses, such as correlation analyses with other measures.

The relationship among age, the general frequency of alcohol/music consumption, tendency to use alcohol/music when experiencing negative emotions, subscale scores (Z-scores) in the DMQ-RJ and B-MMR-J, subscale scores in the CERQ-short-RJ and TIPI-J, and PSS total score were confirmed by calculating correlation coefficients and partial correlation coefficients.

To examine whether the data on the tendency to use alcohol/music when experiencing negative emotions reflect conscious behavior for the purpose of emotion regulation, the study calculated partial correlation coefficients among the tendency for each behavior when experiencing negative emotions and the subscale scores of the CERQ-short-RJ. The control variables were gender, self-perceived ability to metabolize alcohol, and the other eight strategies of the CERQ-short-RJ (excluding the focal strategy). The reasons of selecting these control variables were as follows: (1) This study suggested that gender and self-perceived ability to metabolize alcohol tend to influence alcohol use tendencies during negative emotions (Table 2 and Supplementary Table S2); (2) Garnefski et al.^22^ reported that all nine subscales of the CERQ were positively correlated with depression and anxiety. However, when controlling for intercorrelations among strategies in partial correlation analysis, the results aligned with the theoretical expectations (e.g., rumination, a maladaptive strategy, was positively correlated with depression and anxiety); (3) Sakakibara^36^ conducted a similar partial correlation analysis with Japanese participants and confirmed the positive correlation between rumination and depression.

For a deeper understanding of the effects of age on music use in the context of emotion regulation, we conducted a serial multiple mediation analysis using Model 6 of the PROCESS macro for SPSS (Version 5.0; Hayes^37^). The analysis examined whether the effect of the independent variable (X: age) on the dependent variable (Y: diversion (B-MMR-J)) was mediated by three sequential mediators (M1: TIPI-J, M2: CERQ-short-RJ, M3: PSS-J). The order of mediating variables was theoretically set based on previous studies^38,39^. Subsequently, specific subscales were selected as M1 and M2, based on significant correlations with X, M3, or Y, and their effect sizes. Bootstrapping with 5,000 resamples was used to estimate the indirect effects and to generate bias-corrected 95% confidence intervals (CIs), considering the large number of mediating variables. Standardized coefficients (β) are reported for interpretability.

For all parametric analyses, we verified distributional assumptions primarily through visual diagnostics. Specifically, we inspected Q–Q plots for group-wise scores (Welch’s *t*-test) and standardized residuals (correlation and partial correlation models). Given the large sample size (*N* = 1279), interpretation relied on visual assessment. Robustness was examined via Spearman correlations for bivariate and partial associations, Welch’s *t*-test (already robust to variance inequality) with bootstrap 95% CIs (5000 resamples) for mean differences, and bias-corrected bootstrap CIs for mediation analyses (as described above). Representative Q–Q plots and summary tables for these analyses are provided in Supplementary Tables S3–S4 and Supplementary Fig. S1.

## Results

### Age differences in the general frequency of alcohol/music consumption

Table 1 presents the results of the correlation analysis between age, self-perceived ability to metabolize alcohol, and the general frequencies of drinking alcoholic beverages and listening to music among nonproblem drinkers. The results demonstrated that the number of days for drinking alcohol beverages (number of days in all contexts: total in the alone and not-alone contexts) was significantly positively correlated with age (*r* = 0.11, *p* < 0.001) (Supplementary Fig. S2). In contrast, the number of days that they listened to music in the past 30 days was significantly negatively correlated with age (*r* =  − 0.19, *p* < 0.001) (Supplementary Fig. S3).

**Table 1:** Pearson’s correlation coefficients between age and general frequency of alcohol/music consumption.

		*N*	*M* (*SD*)	1	2	3	4	5	6
1	Age	1279	40.20 (9.94)	–					
2	Self-perceived ability to metabolize alcohol	1223	2.56 (1.18)	− .07*	–				
	Frequency of drinking alcoholic beverages	1279							
3	All contexts		4.78 (8.10)	.11**	.32**	–			
4	Alone context		3.25 (6.92)	.10**	.25**	.84**	–		
5	Not-alone context (All − Alone)		1.53 (4.41)	.06*	.20**	.52**	− .03	–	
6	Frequency of listening to music	1275	13.67 (11.26)	− .19**	.04	.03	.01	.03	–

Data analysis was limited to respondents who scored < 8 in the Alcohol Use Disorders Identification Test.

Each frequency was self-reported as the number of days (0–30) in which each behavior was performed in the past 30 days.

Out of the 1279 participants, 56 never consumed alcoholic beverages.

Four missing values were noted for data on the frequency of listening to music.

**p < .01, *p < .05

### Age differences in the tendency to use alcohol/music when experiencing negative emotions

Table 2 provides the results of the correlation analysis between age, self-perceived ability to metabolize alcohol, and the tendency of displaying the following behaviors when experiencing negative emotions: drinking alcoholic beverages without listening to music, drinking alcoholic beverages while listening to music, and listening to music without drinking alcoholic/caffeinated beverages. The results indicated that the tendency of listening to music without drinking alcoholic/caffeinated beverages when experiencing negative emotions was significantly negatively correlated with age (*r* =  − 0.13, *p* < 0.001). Conversely, the study found no significant correlation between age and the remaining tendencies. Furthermore, the relationships between each tendency were significantly positively correlated even when age was controlled (Table 3).

**Table 2:** Pearson’s correlation coefficients between age and tendency to use alcohol/music while experiencing negative emotions.

		*N*	*M* (*SD*)	1	2	3	4	5
1	Age	1279	40.20 (9.94)	–				
2	Self-perceived ability to metabolize alcohol	1223	2.56 (1.18)	− .07*	–			
	Tendency of doing each behavior when experiencing negative emotions	1275						
3	Drinking alcoholic beverages without listening to music		1.81 (1.09)	.03	.35**	–		
4	Drinking alcoholic beverages with listening to music		1.47 (0.81)	.02	.25**	.68**	–	
5	Listening to music without drinking alcoholic/caffeinated beverages		2.41 (1.13)	− .13**	.00	.29**	.36**	–

Data analysis was limited to respondents who scored < 8 in the Alcohol Use Disorders Identification Test.

Each tendency was self-reported on a five-point Likert scale (from 1 = *Never do it* to 5 = *Always do it*).

Four missing values were noted for data on each tendency.

**p < .01, *p < .05

**Table 3:** Partial correlations between general frequency of alcohol/music consumption and tendency to use alcohol/music while experiencing negative emotions.

		1	2	3	4	5	6	7
	Frequency of drinking alcoholic beverages							
1	All contexts	–						
2	Alone context	.82**	–					
3	Not-alone context (All − Alone)	.49**	− .09**	–				
4	Frequency of listening to music	.04	.02	.04	–			
	Tendency of doing each behavior when experiencing negative emotions							
5	Drinking alcoholic beverages without listening to music	.45**	.41**	.16**	.17**	–		
6	Drinking alcoholic beverages with listening to music	.38**	.34**	.13**	.19**	.65**	–	
7	Listening to music without drinking alcoholic/caffeinated beverages	.11**	.10**	.04	.55**	.33**	.39**	–

Data analysis was limited to respondents who scored < 8 in the Alcohol Use Disorders Identification Test.

Control variables were age, gender, and self-perceived ability to metabolize alcohol.

Missing values and data from those who had never drunk alcohol were excluded from the analysis.

*N* = 1219, **p < .01

### Age differences in motives for drinking alcohol beverages and strategies for mood regulation through music

As described in “Age differences in the general frequency of alcohol/music consumption”, significant correlations existed between age and the frequencies of drinking alcohol beverages and listening to music. In addition, significant positive correlations were observed between the frequency of drinking alcoholic beverages and subscale scores in the Japanese version of the Drinking Motives Questionnaire-Revised (DMQ-RJ) and between the frequency of listening to music and subscale scores in the Japanese version of the Brief Music in Mood Regulation Scale (B-MMR-J) (Supplementary Table S5 and S6). This study focused on age differences in the motives/strategies underlying alcohol/music consumption without the influence of frequency. Therefore, item scores in the DMQ-RJ and B-MMR-J were standardized within each participant and within each scale (within-person Z-scores), using that participant’s own mean and standard deviation across all items of the respective scale, to allow interpretation of subscale scores relative to each participant’s overall response pattern (see “Statistical analysis” for details). These standardized subscale scores were then included in the correlation analysis with age (Table 4). The results demonstrated that levels of conformity (DMQ-RJ) were significantly negatively correlated with age (*r* =  − 0.14, *p* < 0.001). Additionally, the levels of social and enhancement (DMQ-RJ) were significantly positively correlated with age (*r* = 0.10, *p* = 0.002; *r* = 0.09, *p* = 0.006). On the other hand, the level of diversion (B-MMR-J) was significantly negatively correlated with age (*r* =  − 0.09, *p* = 0.003), and the level of mental work (B-MMR-J) was significantly positively correlated with age (*r* = 0.09, *p* = 0.003). These correlations are significant at α = 0.01 and slightly below the indicative effect-size benchmark (|*r*|≥ 0.10, which are reference values rather than strict cut-offs); although small, they may still be noteworthy in the context of our research question. For other motives/strategies, the study noted no significant correlation with age.

**Table 4:** Pearson’s correlation coefficients between age and the subscale scores of DMQ-R and B-MMR.

		*N*	*M* (*SD*)	1	2	3	4	5	6	7	8	9	10	11	12
1	Age	1279	40.20 (9.94)	–											
	DMQ-RJ (Z-score)	926													
2	Social		1.86 (3.16)	.10**	–										
3	Coping		− 0.44 (2.89)	− .05	− .56**	–									
4	Enhancement		1.50 (2.69)	.09**	− .37**	− .09**	–								
5	Conformity		− 2.92 (2.77)	− .14**	− .20**	− .32**	− .46**	–							
	B-MMR-J (Z-score)	1151													
6	Entertainment		0.80 (2.27)	.04	.20**	− .15**	.00	− .08*	–						
7	Revival		0.33 (1.56)	.01	− .05	.05	.02	− .01	− .12**	–					
8	Strong Sensation		0.91 (1.85)	.06*	.13**	− .15**	.09*	− .08*	− .07*	− .25**	–				
9	Diversion		− 0.19 (1.50)	− .09**	− .17**	.17**	− .04	.04	− .32**	− .01	− .28**	–			
10	Discharge		− 2.34 (2.05)	− .06*	− .10**	.01	− .07*	.17**	− .20**	− .26**	− .20**	− .19**	–		
11	Mental Work		0.35 (1.47)	.09**	− .01	.02	.03	− .05	− .34**	− .12**	− .06	− .03	− .21**	–	
12	Solace		0.14 (1.56)	− .05	− .09**	.14**	− .02	− .02	− .38**	− .08**	− .27**	.12**	− .16**	.05	–

Data analysis was limited to respondents who scored < 8 in the Alcohol Use Disorders Identification Test.

*DMQ-RJ* Japanese version of the Drinking Motives Questionnaire-Revised, *B-MMR-J* Japanese version of the Brief Music in Mood Regulation Scale.

The Z-scores for DMQ-RJ could not be calculated for 353 participants.

The Z-scores for B-MMR-J could not be calculated for 128 participants.

**p < .01, *p < .05

### Age differences in cognitive emotion regulation strategies

Table 5 depicts the result of the correlation analysis between age and the subscale scores of the Revised Japanese version of the Cognitive Emotion Regulation Questionnaire (CERQ-short-RJ). The results implied that the subscale scores for refocus on planning and positive reappraisal were significantly positively correlated with age (*r* = 0.14, *p* < 0.001; *r* = 0.11, *p* < 0.001). These results indicated that older nonproblem drinkers may generally cope and think more adaptively with negative or unpleasant events compared with younger nonproblem drinkers.

**Table 5:** Pearson’s correlation coefficients between age and cognitive emotion regulation strategies.

		*M* (*SD*)	1	2	3	4	5	6	7	8	9	10
1	Age	40.20 (9.94)	–									
	CERQ-short-RJ											
2	Self-blame	6.39 (1.90)	− .06*	–								
3	Acceptance	6.75 (1.89)	.03	.59**	–							
4	Rumination	6.47 (1.98)	− .06*	.52**	.48**	–						
5	Positive refocusing	5.74 (2.00)	− .03	.18**	.27**	.12**	–					
6	Refocus on planning	6.90 (1.89)	.14**	.36**	.55**	.41**	.33**	–				
7	Positive reappraisal	5.99 (2.05)	.11**	.30**	.47**	.22**	.50**	.60**	–			
8	Putting into perspective	5.72 (1.82)	.03	.34**	.46**	.28**	.52**	.41**	.55**	–		
9	Catastrophizing	6.14 (1.96)	− .03	.50**	.46**	.72**	.12**	.44**	.27**	.31**	–	
10	Other-blame	5.00 (1.84)	− .07*	− .07*	.03	.27**	.25**	.12**	.12**	.22**	.31**	–

Data analysis was limited to respondents who scored < 8 in the Alcohol Use Disorders Identification Test.

*CERQ-short-RJ* Revised Japanese version of the Cognitive Emotion Regulation Questionnaire.

*N* = 1279, **p < .01, *p < .05

### Mediation effect of extraversion, positive reappraisal, and perceived stress between age and diversion strategy through music

**Fig. 1:**
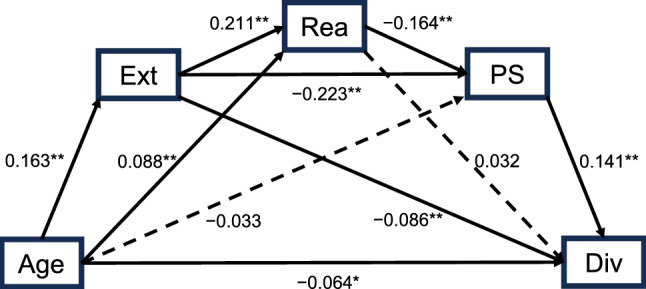
Mediation effect path of extraversion, positive reappraisal, and perceived stress between age and diversion strategy through music. Numbers are presented with standardized coefficients. **p < 0.01, *p < 0.05. *Ext* extraversion, *Rea* positive reappraisal, *PS* perceived stress, *Div* diversion strategy through music.

Diversion subscale, which was significantly negatively correlated with age (Table 4), was most strongly associated with music use tendencies when experiencing negative emotions among the B-MMR-J subscales (*r* = 0.26, *p* < 0.001) (Supplementary Table S7). Thus, to gain a deeper understanding of the relationship between age and diversion strategy through music, sequential mediation analysis was conducted. As mediator variables, not only cognitive emotion regulation strategy, but also personality and stress, which were reported to be associated with emotion regulation, were analyzed^38,39^ (see “Statistical analysis” and Supplementary Table S8 for details). The analysis revealed the total effect of age (X) on diversion strategy through music (Y) was significant, β =  − 0.087, *SE* = 0.029, *p* = 0.003, R^2^ = 0.008, and the significant indirect effect of X on Y through the three mediators in sequence (X → extraversion (M1) → positive reappraisal (M2) → perceived stress (M3) → Y), β =  − 0.001, *SE* = 0.000, 95% CI [− 0.002, − 0.000]. The significant specific indirect effects were as follows: (1) X → M1 → Y: β =  − 0.014, *SE* = 0.005, 95% CI [− 0.025, − 0.004]; (2) X → M1 → M3 → Y: β =  − 0.005, *SE* = 0.002, 95% CI [− 0.009, − 0.002]; (3) X → M2 → M3 → Y: β =  − 0.002, *SE* = 0.001, 95% CI [− 0.004, − 0.001]. The total indirect effect was significant, β =  − 0.023, *SE* = 0.008, 95% CI [− 0.039, − 0.007]. The direct effect of X on Y, controlling for the mediators, was β =  − 0.064, *SE* = 0.030, *p* = 0.029, indicating that partial mediation (Table 6 and Fig. 1).

**Table 6:** Standardized regression coefficients, standard error, and 95% confidence intervals for each path.

Effect	Path	β	*SE*	*p*	95% CI
Total effect	X: Age → Y: Div	− 0.087	0.029	0.003	[− 0.145, − 0.029]
Direct effect	X: Age → Y: Div (controlling for M1: Ext, M2: Rea, and M3: PS)	− 0.064	0.030	0.029	[− 0.122, − 0.007]
Indirect effect 1	X: Age → M1: Ext → Y: Div	− 0.014	0.005	–	[− 0.025, − 0.004]
Indirect effect 2	X: Age → M2: Rea → Y: Div	0.003	0.003	–	[− 0.003, 0.010]
Indirect effect 3	X: Age → M3: PS → Y: Div	− 0.005	0.004	–	[− 0.014, 0.003]
Indirect effect 4	X: Age → M1: Ext → M2: Rea → Y: Div	0.001	0.001	–	[− 0.001, 0.003]
Indirect effect 5	X: Age → M1: Ext → M3: PS → Y: Div	− 0.005	0.002	–	[− 0.009, − 0.002]
Indirect effect 6	X: Age → M2: Rea → M3: PS → Y: Div	− 0.002	0.001	–	[− 0.004, − 0.001]
Indirect effect 7	X: Age → M1: Ext → M2: Rea → M3: PS → Y: Div	− 0.001	0.000	–	[− 0.002, − 0.000]

*Ext* extraversion, *Rea* positive reappraisal, *PS* perceived stress, *Div* diversion strategy through music.

The statistical significance of each indirect effect was determined based on whether the 95% CI excluded zero (considering the large number of mediating variables, the confidence interval was set at 95% in this analysis).

## Discussion

The current study intended to comprehensively explore age differences in alcohol/music consumption, especially in the context of emotion regulation. To the best of our knowledge, this study is the first to use the Drinking Motives Questionnaire-Revised (DMQ-R) and Brief Music in Mood Regulation Scale (B-MMR) simultaneously. The target of data analysis was limited to 1279 participants with Alcohol Use Disorders Identification Test (AUDIT) scores of < 8, given our interest in age difference in the use or combination of alcohol and music when experiencing negative emotions and the relationship among these behaviors, in individuals without indications of problematic drinking.

Regarding age differences in the general frequency of alcohol/music consumption, younger nonproblem drinkers displayed lower frequencies of drinking alcoholic beverages and higher frequencies of listening to music. These results indicate that trends in the drinking rates of Japanese people by age group^2^, including problem drinkers, and the relationship between age and music consumption reported by Chamorro-Premuzic et al.^12^, are maintained even when limited to people who do not fall under the category of problem drinkers.

Second, the results implied that no age-related differences in the tendency of drinking alcoholic beverages, whether with or without listening to music, when experiencing negative emotions. This aspect may be related to the fact that both behaviors were rarely chosen as methods to regulate emotions (the average tendency scores on a five-point Likert scale [1 = *Never do it*, 5 = *Always do it*] were less than 2.00 for both behaviors). Alternatively, younger nonproblem drinkers displayed higher tendencies of listening to music without drinking alcoholic/caffeinated beverages when experiencing negative emotions. These data imply that older nonproblem drinkers use other means of addressing negative emotions than drinking alcoholic beverages or listening to music, or they can cognitively resolve their emotions without resorting to specific actions. In addition, when general music listening frequency was controlled, the association between age and the tendency to listen to music during negative emotions became no longer statistically significant. This suggests that the age-related decline in listening to music for emotion regulation may largely reflect the age-related decline in general music listening frequency. It might be that frequent familiar behaviors are easily recognized as means of emotion regulation. On the other hand, there is concern that these tendency data, obtained using the protocol described in “Tendency of drinking alcoholic beverages/listening to music when experiencing negative emotions”, might not reflect conscious behavior to regulate emotions. For example, partial correlation analyses between the tendencies of alcohol use and music use when experiencing negative emotions and subscale scores in the Revised Japanese version of the Cognitive Emotion Regulation Questionnaire (CERQ-short-RJ) indicated that these tendencies were not/weakly related to emotion regulation (Supplementary Table S9). However, among the subscales of DMQ-R and B-MMR, diversion (B-MMR) showed the strongest positive correlation with tendency of listening to music without drinking alcoholic/caffeinated beverages when experiencing negative emotions. Diversion was not correlated with tendency of drinking alcoholic beverages with/without listening to music, and tendency of drinking alcoholic beverages without listening to music showed a positive correlation with coping (DMQ-R) (Supplementary Table S7). Therefore, although aforementioned concern couldn’t be denied completely, the measurement method of “Tendency of Drinking Alcoholic Beverages/Listening to Music When Experiencing Negative Emotions” in this study (“Tendency of drinking alcoholic beverages/listening to music when experiencing negative emotions”) can be considered to have a certain degree of validity as a measure of conscious behavior to regulate emotions. Additionally, in both cases when age was controlled or not, alcohol use tendencies and music use tendencies when experiencing negative emotions were positively correlated. These results challenge the assumption that if alcohol and music function as alternative rather than complementary means of emotion regulation, their frequency of use in the context of emotion regulation should show a negative correlation.

Third, regarding age differences in the motives for drinking alcohol beverages, the most notable difference was in the subscale score in conformity, which was higher for younger nonproblem drinkers. This result implied that younger nonproblem drinkers exhibit a stronger motive to fit in with others or avoid social rejection related to drinking. In Erikson’s stages of psychosocial developmental theory, the developmental tasks and crises in young adulthood are related to intimacy versus isolation^40,41^. Therefore, the age difference in drinking motive observed in the current study may be related to this concept. Regarding age differences in the strategies for mood regulation through music, younger nonproblem drinkers showed a higher subscale score in diversion and a lower in mental work than older nonproblem drinkers. In particular, diversion was most strongly associated with music use tendencies when experiencing negative emotions among the subscales. Additionally, the results of mediation analysis suggested that the higher diversion scores among younger nonproblem drinkers may be related to their lower extraversion, lower positive reappraisal, and higher stress levels. Based on these results, the fact that older nonproblem drinkers showed higher subscale score on mental work (B-MMR) including the item “Music has helped me to get through difficult experiences” may reflect the possibility that they were recalling their youthful experiences of listening to music while experiencing negative emotions due to social factors related with psychosocial tasks and psychosocial crises (intimacy vs. isolation).

### Limitations and further research

The first limitation of this study is that it was conducted only on Japanese respondents. Given differences between countries in terms of alcohol metabolism^42^, musical preference^43^, and personality^44,45^, including the relationship between depression and cognitive emotion regulation^46^, a possibility exists that the age differences observed in the current study among nonproblem drinkers in Japan may not be identified among non-Japanese people. For example, in this study, we found that social and enhancement of the DMQ-R were positively correlated with age. However, based on the findings reported by Mackinnon et al.^8^ regarding the relationship between cultural differences in individualism versus collectivism and drinking motives, unlike Japan, which is a collectivist country^47^, in other individualistic countries, social and enhancement motives may be more pronounced even among younger individuals, potentially resulting in the absence of age differences. Additionally, we found that diversion of the B-MMR was negatively correlated with age in this study. These findings may be interpreted in light of Japanese cultural practices. Middle-aged adults may have greater access to culturally sanctioned emotional outlets such as workplace drinking, while younger adults may be more engaged in emotionally expressive practices like karaoke or digital music streaming. These distinctions underscore the importance of culturally contextualized approaches when studying emotion regulation strategies.

Second, this study is cross-sectional in nature, and the older participants were not queried about their drinking and music experiences when they were young. Thus, whether the older participants previously exhibited the same traits as the younger participants remains unknown.

The third limitation is whether the data on the tendency to use alcohol/music when experiencing negative emotions, which were obtained using the instructions described in “Tendency of drinking alcoholic beverages/listening to music when experiencing negative emotions”, reflected behavior that consciously targeted emotion regulation is unclear. Improvements in the instructions (e.g., rewording the item, “When you are experiencing negative emotions, do you sometimes do the following things to change negative emotions?”) are required to enable an in-depth examination of the relationship between alcohol/music consumption and emotion regulation for each age group. Furthermore, when responding to the instruction “when experiencing negative emotions”, participants may have felt confused about which emotions to consider. Future surveys should pre-specify exemplar emotions (e.g., sadness, anger) or provide clarifying examples to reduce potential ambiguity.

Finally, although several associations reached statistical significance, their magnitudes were small. This pattern might be regarded as consistent with the diverse, multi-determined, and complex nature of psycho-social phenomena. We therefore interpret the findings cautiously and note that confirming reproducibility in independent samples may be warranted.

## Conclusions

This study explored age differences in alcohol/music consumption, especially in the context of emotion regulation, among Japanese nonproblem drinkers. Among alcohol consumption/combined alcohol and music consumption/music consumption when experiencing negative emotions, age difference was observed only in music consumption, with younger individuals exhibiting a stronger tendency to consume music in that context. This was suggested to be due to lower extraversion and lower positive reappraisal strategies among younger people, which were associated with their higher stress levels, leading them to use music as a means of diversion. Furthermore, people who consume alcohol to regulate emotions showed higher tendency to consume alcohol in combination with music or listen to music in the context of emotion regulation, which was the case for both younger and older participants. This preliminary study contributes to the development of research that elucidates how the concrete means for emotion regulation change over the course of psychosocial development.

## Supplementary Information

Supplementary Information.

## Data Availability

The datasets generated and/or analyzed during this study are available from the corresponding author on reasonable request.

## References

[1] Watanabe, S. Music and drinking behaviour. *J. Brew. Soc. Jpn.* **86**, 640–644 (1991).

[2] Japanese Ministry of Health, Labour and Welfare. Alcohol and health. *E-health Net*. https://kennet.mhlw.go.jp/information/information/alcohol/a-06-001 (n.d.).

[3] Imada, S., Furumitsu, I. & Izu, H. Development of the five factor Drinking Motive Questionnaire for Japanese (DMQ-J). *Hiroshima Shudo Univ. J.* **57**, 153–162 (2017).

[4] Okada, Y. Motives for drinking and problem drinking in middle-aged people: Examination of DMQ-R and AUDIT based on gender. *J. Health Psychol. Res.* **32**, 55–63 (2020).

[5] Cooper, M. L. Motivations for alcohol use among adolescents: Development and validation of a four-factor model. *Psychol. Assess.* **6**, 117–128 (1994).

[6] Gilson, K. M. et al. Validation of the Drinking Motives Questionnaire (DMQ) in older adults. *Addict. Behav.* **38**, 2196–2202 (2013). 23454884 10.1016/j.addbeh.2013.01.021

[7] Cooper, M. L., Kuntsche, E., Levitt, A., Barber, L. L. & Wolf, S. Motivational models of substance use: A review of theory and research on motives for using alcohol, marijuana, and tobacco. In *The Oxford Handbook of Substance Use and Substance Use Disorders* (ed. Sher, K. J.) 375–421 (Oxford University Press, 2015).

[8] Mackinnon, S. P. et al. Cross-cultural comparisons of drinking motives in 10 countries: Data from the DRINC project. *Drug Alcohol Rev.* **36**, 721–730 (2017). 28337801 10.1111/dar.12464

[9] Hofstede, G., Hofstede, G. J. & Minkov, M. *Cultures and Organizations: Software of the Mind*, 3rd ed. (Mcgraw-Hill, 2010).

[10] Ben-Ari, E. Sake and “Space Time”: Culture, Organization and Drinking in Japanese Firms. *Senri Ethnol. Stud.* **64**, 89–100 (2003).

[11] Mitsui, T. & Hosokawa, S. *Karaoke Around the World: Global Technology, Local Singing* (Routledge, 1998).

[12] Chamorro-Premuzic, T., Swami, V. & Cermakova, B. Individual differences in music consumption are predicted by uses of music and age rather than emotional intelligence, neuroticism, extraversion or openness. *Psychol. Music* **40**, 285–300 (2010).

[13] Chamorro-Premuzic, T. & Furnham, A. Personality and music: Can traits explain how people use music in everyday life?. *Br. J. Psychol.* **98**, 175–185 (2007). 17456267 10.1348/000712606X111177

[14] Gross, J. J. The emerging field of emotion regulation: An integrative review. *Rev. Gen. Psychol.* **2**, 271–299 (1998).

[15] Chong, H. J., Kim, H. J. & Kim, B. Scoping review on the use of music for emotion regulation. *Behav. Sci.* **14**, 793 (2024). 39336008 10.3390/bs14090793 PMC11428991

[16] Saarikallio, S. H. Music in mood regulation: Initial scale development. *Mus. Sci.* **12**, 291–309 (2008).

[17] Saarikallio, S. Development and validation of the Brief Music in Mood Regulation scale (B-MMR). *Music Percept.* **30**, 97–105 (2012).

[18] Ansani, A., Mallia, L. & Saarikallio, S. Music in Mood Regulation Brief scale (B-MMR): Italian version and insights on musical preferences, expertise, and reward experiences. *Music Percept.* **42**, 389–405 (2025).

[19] Shoda, H., Yasuda, S., Nakahara, J., Tabei, K. & Isaka, T. Development of the Japanese version of the Brief Music Mood Regulation Scale and evaluation of its reliability and validity. *Jpn. J. Psychol.* **90**, 398–407 (2019).

[20] Bailly, N. et al. Effects of gender and age on cognitive emotional regulation. *Curr. Psychol.* **42**, 27228–27231 (2023).

[21] Garnefski, N., Kraaij, V. & Spinhoven, P. Negative life events, cognitive emotion regulation and emotional problems. *Pers. Individ. Differ.* **30**, 1311–1327 (2001).

[22] Garnefski, N. & Kraaij, V. Relationships between cognitive emotion regulation strategies and depressive symptoms: A comparative study of five specific samples. *Pers. Individ. Differ.* **40**, 1659–1669 (2006).

[23] Persson, J. & Magnusson, P. H. Early intervention in patients with excessive consumption of alcohol: A controlled study. *Alcohol* **6**, 403–408 (1989). 2573364 10.1016/0741-8329(89)90011-6

[24] Saunders, J. B. & Foulds, K. Brief and early intervention: experience from studies of harmful drinking. *Aust. N. Z. J. Med.* **22**, 224–230 (1992). 1530550 10.1111/j.1445-5994.1992.tb02818.x

[25] Şahin, F. & Çetin, F. The mediating role of general self-efficacy in the relationship between the Big Five personality traits and perceived stress: A weekly assessment study. *Psychol Stud.* **62**, 35–46 (2017).

[26] Hussong, A. M. Social influences in motivated drinking among college students. *Psychol Addict Behav.* **17**, 142–150 (2003). 12814278 10.1037/0893-164x.17.2.142

[27] Hiro, H. *WHO/AUDIT the Alcohol Use Disorders Identification Test* (Japanese Version) (Chiba Test Center, 2000).

[28] Babor, T. F., Higgins-Biddle, J. C., Saunders, J. B. & Monteiro, M. G. AUDIT: the alcohol use disorders identification test: guidelines for use in primary health care. 2nd ed. *World Health Organization* (2001).

[29] Urano, Y., Kobayashi, R. & Sakakibara, R. Revision and validation of the Japanese-version cognitive emotion regulation questionnaire: psychometric properties and measurement invariance across gender. *Cogent Psychol.* **9**, 2064790. 10.1080/23311908.2022.2064790 (2022).

[30] Sakakibara, R. The usage tendency of and the relation with psychological health of cognitive emotion regulation: Development of Japanese-version Cognitive Emotion Regulation Questionnaire and focus on the effects of negative emotional intensity. *Jpn. J. Res. Emot.* **23**, 46–58 (2015).

[31] Gosling, S. D., Rentfrow, P. J. & Swann, W. B. Jr. A very brief measure of the Big-Five personality domains. *J. Res. Pers.* **37**, 504–528 (2003).

[32] Oshio, A., Abe, S. & Cutrone, P. Development, reliability, and validity of the Japanese version of Ten Item Personality Inventory (TIPI-J). *Jpn. J. Pers.* **21**, 40–52 (2012).

[33] Cohen, S., Kamarck, T. & Mermelstein, R. A global measure of perceived stress. *J. Health Soc. Behav.* **24**, 385–396 (1983). 6668417

[34] Sumi, K. Reliability and validity of the Japanese version of the Perceived Stress Scale. *Jpn. J. Health Psychol.* **19**, 44–53 (2006).

[35] Mizumoto, A. & Takeuchi, O. Basics and considerations for reporting effect sizes in research papers. *Stud. Engl. Lang. Teach.* **31**, 57–66 (2008).

[36] Sakakibara, R. How does cognitive appraisal moderate the relationship between cognitive emotion regulation strategies and psychological health?. *Jpn. J. Soc. Psychol.* **32**, 163–173 (2017).

[37] Hayes, A. F. *Introduction to Mediation, Moderation, and Conditional Process Analysis: A Regression-Based Approach.* 3rd ed. (Guilford Press, 2022).

[38] Hughes, D. J., Kratsiotis, I. K., Niven, K. & Holman, D. Personality traits and emotion regulation: A targeted review and recommendations. *Emotion* **20**, 63–67 (2020). 31961180 10.1037/emo0000644

[39] Martin, R. C. & Dahlen, E. R. Cognitive emotion regulation in the prediction of depression, anxiety, stress, and anger. *Pers Individ Dif.* **39**, 1249–1260 (2005).

[40] Erikson, E. H. *Childhood and Society* (Norton, 1950).

[41] Erikson, E. H. *Childhood and Society* 2nd edn. (Norton, 1963).

[42] Agarwal, D. P. & Goedde, H. W. Alcohol metabolism: biochemistry and genetic variations. In *Alcohol Metabolism, Alcohol Intolerance, and Alcoholism* (eds. Agarwal, D. P. & Goedde, H. W.) 6–51 (Springer, 1990).

[43] LeBlanc, A., Jin, Y. C., Stamou, L. & McCrary, J. Effect of age, country, and gender on music listening preferences. *Bull. Counc. Res. Music Educ.* **141**, 72–76 (1999).

[44] Kajonius, P. & Mac Giolla, E. Personality traits across countries: Support for similarities rather than differences. *PLoS ONE* **12**, e0179646 (2017). 28622380 10.1371/journal.pone.0179646 PMC5473578

[45] McCrae, R. R. & Terracciano, A. Personality profiles of cultures: Aggregate personality traits. *J. Pers. Soc. Psychol.* **89**, 407–425 (2005). 16248722 10.1037/0022-3514.89.3.407

[46] Sakakibara, R. & Kitahara, M. The relationship between Cognitive Emotion Regulation Questionnaire (CERQ) and depression, anxiety: Meta-analysis. *Jpn. J. Psychol.* **87**, 179–185 (2016). 10.4992/jjpsy.87.15302 27476268

[47] Markus, H. R. & Kitayama, S. Culture and the self: Implications for cognition, emotion, and motivation. *Psychol Rev.* **98**, 224–253 (1991).

