# Supplementary material for: Age differences in alcohol and music consumption among Japanese nonproblem drinkers
Source: Sci Rep. 2025 Dec 7;15:43432. doi: 10.1038/s41598-025-26809-0 (PMC12690111; doi:10.1038/s41598-025-26809-0)
Supplement: Supplementary file 1 — Supplementary Information. [file 41598_2025_26809_MOESM1_ESM.pdf]

Age differences in alcohol and music consumption among Japanese nonproblem drinkers

Naoki Kato[1,3 *], Shunji Oshima[2], Katsumi Watanabe[3]

[1] Core Technology Laboratories, Asahi Quality & Innovations, Ltd., 1-21 Midori 1-chome, Moriya, Ibaraki 302-0106, Japan

[2] Sustainable Technology Laboratories, Asahi Quality & Innovations, Ltd., 1-21 Midori 1-chome, Moriya, Ibaraki 302-0106, Japan

[3] Faculty of Science and Engineering, Waseda University, 3-4-1 Okubo, Shinjuku-ku, Tokyo 169-8555, Japan

*Corresponding author.

E-mail address: naoki.kato@asahi-qi.co.jp (N. Kato).

Supplementary Table S1. AUDIT scores by gender and age range

		***M* (*SD*)**	**0–7**	**8–40**	
			***N* (%)**		**χ2 and Cramer's *V***
**All**		4.10 (5.40)	1279 (83.59)	251 (16.41)	
**Gender**	**Male**	5.23 (6.18)	514 (33.59)	155 (10.13)	$\chi2_{(1)} = 39.66$
	**Female**	3.23 (4.52)	765 (50.00)	96 (6.27)	Cramer's *V* = 0.16**
**Age range**	**20s**	3.39 (5.20)	207 (13.53)	36 (2.35)	$\chi2_{(3)} = 7.25$
	**30s**	3.89 (4.88)	445 (29.08)	69 (4.51)	Cramer's *V* = 0.07
	**40s**	4.46 (5.64)	349 (22.81)	81 (5.29)	
	**50s**	4.49 (5.89)	278 (18.17)	65 (4.25)	

Note. ** $p < .01$

AUDIT = Alcohol Use Disorders Identification Test

Supplementary Table S2. Gender difference in the each item/subscale score

	Male (N = 514)	Female (N = 765)				
	M (*SD*)		*t* test (*df*)	*p*	Cohen's *d*	95% CI
**Age**	39.02 (9.34)	40.99 (10.26)	−3.57 (1166.98)	0.000	−0.20	[−0.31, −0.09]
**AUDIT / Total score**	2.55 (2.34)	1.98 (2.05)	4.68 (1035.18)	0.000	0.27	[0.16, 0.38]
**Self-perceived ability to metabolize alcohol**	2.60 (1.20)	2.53 (1.17)	1.11 (1028.15)	0.266	0.07	[−0.05, 0.18]
**Frequency of drinking alcoholic beverages**						
All contexts	5.05 (8.05)	4.60 (8.12)	0.98 (1106.82)	0.326	0.06	[−0.06, 0.17]
Alone context	3.90 (7.31)	2.81 (6.62)	2.71 (1025.52)	0.007	0.16	[0.05, 0.27]
Not-alone context (All − Alone)	1.15 (3.78)	1.79 (4.77)	−2.64 (1243.19)	0.008	−0.14	[−0.26, −0.03]
**Frequency of listening to music**	14.26 (11.33)	13.28 (11.2)	1.53 (1084.29)	0.127	0.09	[−0.02, 0.20]
**Tendency of doing each behavior when experiencing negative emotions**						
Drinking alcoholic beverages without listening to music	1.88 (1.11)	1.77 (1.08)	1.71 (1071.58)	0.088	0.10	[−0.01, 0.21]
Drinking alcoholic beverages with listening to music	1.49 (0.78)	1.46 (0.83)	0.71 (1142.86)	0.478	0.04	[−0.07, 0.15]
Listening to music without drinking alcoholic/caffeinated beverages	2.41 (1.12)	2.41 (1.15)	0.06 (1112.12)	0.952	0.00	[−0.11, 0.12]
**DMQ-RJ (Z-score)**						
Social	1.68 (3.08)	1.99 (3.21)	−1.50 (870.24)	0.134	−0.10	[−0.23, 0.03]
Coping	−0.33 (2.77)	−0.51 (2.98)	0.97 (882.98)	0.332	0.06	[−0.07, 0.19]
Enhancement	1.45 (2.67)	1.54 (2.70)	−0.55 (858.85)	0.585	−0.04	[−0.17, 0.09]
Conformity	−2.80 (2.78)	−3.02 (2.76)	1.22 (848.74)	0.222	0.08	[−0.05, 0.21]
**B-MMR-J (Z-score)**						
Entertainment	0.65 (2.19)	0.90 (2.32)	−1.83 (1025.98)	0.068	−0.11	[−0.23, 0.01]
Revival	0.47 (1.57)	0.23 (1.55)	2.54 (978.31)	0.011	0.15	[0.04, 0.27]
Strong Sensation	0.82 (1.89)	0.97 (1.82)	−1.32 (960.85)	0.188	−0.08	[−0.20, 0.04]
Diversion	−0.10 (1.50)	−0.26 (1.51)	1.74 (993.38)	0.082	0.10	[−0.01, 0.22]
Discharge	−2.28 (1.97)	−2.38 (2.10)	0.86 (1032.95)	0.390	0.05	[−0.07, 0.17]
Mental Work	0.36 (1.45)	0.35 (1.48)	0.17 (1000.79)	0.867	0.01	[−0.11, 0.13]
Solace	0.07 (1.51)	0.19 (1.59)	−1.29 (1020.75)	0.196	−0.08	[−0.19, 0.04]
**CERQ-short-RJ**						
Self-blame	6.30 (1.81)	6.46 (1.96)	−1.54 (1157.49)	0.124	−0.09	[−0.20, 0.03]
Acceptance	6.58 (1.85)	6.86 (1.91)	−2.56 (1126.61)	0.011	−0.15	[−0.26, −0.03]
Rumination	6.18 (1.91)	6.67 (2.00)	−4.44 (1132.53)	0.000	−0.25	[−0.36, −0.14]
Positive refocusing	5.72 (1.89)	5.76 (2.07)	−0.28 (1165.01)	0.777	−0.02	[−0.13, 0.10]
Refocus on planning	6.73 (1.83)	7.01 (1.93)	−2.57 (1139.65)	0.010	−0.14	[−0.26, −0.03]
Positive reappraisal	5.93 (1.96)	6.03 (2.11)	−0.90 (1152.13)	0.367	−0.05	[−0.16, 0.06]
Putting into perspective	5.72 (1.75)	5.73 (1.87)	−0.08 (1148.71)	0.936	0.00	[−0.12, 0.11]
Catastrophizing	6.02 (1.84)	6.21 (2.04)	−1.73 (1172.43)	0.083	−0.10	[−0.21, 0.01]
Other-blame	5.21 (1.83)	4.85 (1.84)	3.42 (1105.67)	0.001	0.19	[0.08, 0.31]
**TIPI-J**						
Extraversion	6.76 (2.84)	7.17 (2.97)	−2.48 (1134.97)	0.013	−0.14	[−0.25, −0.03]
Agreeableness	9.25 (2.48)	9.76 (2.49)	−3.59 (1104.14)	0.000	−0.20	[−0.32, −0.09]
Conscientiousness	7.78 (2.93)	8.25 (2.99)	−2.77 (1114.14)	0.006	−0.16	[−0.27, −0.05]
Neuroticism	8.13 (2.83)	8.61 (2.76)	−3.00 (1084.04)	0.003	−0.17	[−0.28, −0.06]
Openness to Experience	7.41 (2.72)	6.93 (2.83)	3.02 (1130.8)	0.003	0.17	[0.06, 0.28]
**PSS-J / Total score**	19.87 (6.35)	20.44 (6.59)	−1.56 (1126.75)	0.118	−0.09	[−0.20, 0.02]

Note. Data analysis was limited to respondents who scored < 8 in the Alcohol Use Disorders Identification Test.

Each frequency was self-reported as the number of days (0-30) in which each behavior was performed within 30 days.

Each tendency was self-reported on a five-point Likert scale (from 1 = *Never do it* to 5 = *Always do it*).

The Z-scores for DMQ-RJ could not be calculated for 117 participants in male and 236 participants in female.

The Z-scores for B-MMR-J could not be calculated for 51 participants in male and 76 participants in female.

There were each four missing values for the data on the frequency of listening to music and tendency of doing each behavior when experiencing negative emotions.

Abbreviations: AUDIT = Alcohol Use Disorders Identification Test, DMQ-RJ = Japanese version of the Drinking Motives Questionnaire-Revised, B-MMR-J = Japanese version of the Brief Music in Mood Regulation Scale, CERQ-short-RJ = Revised Japanese version of the Cognitive Emotion Regulation Questionnaire, TIPI-J = Japanese version of the Ten Item Personality Inventory, PSS-J = Japanese version of the Perceived Stress Scale.

Supplementary Table S3. Bootstrap 95% CIs (BCa, 5,000 resamples) for mean differences from Welch's *t*-tests of gender

	**Mean difference (Male − Female)**	**Bootstrap 95% CI**
**Age**	−1.40	[−2.70, −0.11]
**AUDIT / Total score**	0.43	[0.15, 0.70]
**Self-perceived ability to metabolize alcohol**	0.03	[−0.13, 0.18]
**Frequency of drinking alcoholic beverages**		
All contexts	0.16	[−1.04, 1.36]
Alone context	1.08	[0.00, 2.15]
Not-alone context (All − Alone)	−0.92	[−1.56, −0.28]
**Frequency of listening to music**	0.80	[−0.68, 2.28]
**Tendency of doing each behavior when experiencing negative emotions**		
Drinking alcoholic beverages without listening to music	0.01	[−0.14, 0.17]
Drinking alcoholic beverages with listening to music	−0.01	[−0.13, 0.10]
Listening to music without drinking alcoholic/caffeinated beverages	−0.01	[−0.16, 0.13]
**DMQ-RJ (Z-score)**		
Social	−0.34	[−0.76, 0.09]
Coping	0.21	[−0.17, 0.59]
Enhancement	−0.09	[−0.45, 0.27]
Conformity	0.22	[−0.16, 0.59]
**B-MMR-J (Z-score)**		
Entertainment	−0.44	[−0.74, −0.14]
Revival	0.24	[0.03, 0.45]
Strong Sensation	−0.20	[−0.45, 0.04]
Diversion	0.32	[0.12, 0.52]
Discharge	0.07	[−0.21, 0.35]
Mental Work	0.02	[−0.18, 0.22]
Solace	−0.01	[−0.22, 0.19]
**CERQ-short-RJ**		
Self-blame	−0.14	[−0.37, 0.10]
Acceptance	−0.23	[−0.47, 0.01]
Rumination	−0.53	[−0.78, −0.27]
Positive refocusing	0.05	[−0.20, 0.31]
Refocus on planning	−0.31	[−0.55, −0.07]
Positive reappraisal	−0.09	[−0.35, 0.18]
Putting into perspective	−0.02	[−0.25, 0.21]
Catastrophizing	−0.23	[−0.48, 0.01]
Other-blame	0.38	[0.15, 0.62]
**TIPI-J**		
Extraversion	−0.55	[−0.94, −0.15]
Agreeableness	−0.52	[−0.83, −0.20]
Conscientiousness	−0.36	[−0.75, 0.03]
Neuroticism	−0.59	[−0.95, −0.22]
Openness to Experience	0.33	[−0.03, 0.69]
**PSS-J / Total score**	−0.81	[−1.63, 0.01]

Note. Data analysis was limited to respondents who scored < 8 in the Alcohol Use Disorders Identification Test.

Each frequency was self-reported as the number of days (0-30) in which each behavior was performed within 30 days.

Each tendency was self-reported on a five-point Likert scale (from 1 = *Never do it* to 5 = *Always do it*).

The Z-scores for DMQ-RJ could not be calculated for 117 participants in male and 236 participants in female.

The Z-scores for B-MMR-J could not be calculated for 51 participants in male and 76 participants in female.

There were each four missing values for the data on the frequency of listening to music and tendency of doing each behavior when experiencing negative emotions.

Abbreviations: AUDIT = Alcohol Use Disorders Identification Test, DMQ-RJ = Japanese version of the Drinking Motives Questionnaire-Revised, B-MMR-J = Japanese version of the Brief Music in Mood Regulation Scale, CERQ-short-RJ = Revised Japanese version of the Cognitive Emotion Regulation Questionnaire, TIPI-J = Japanese version of the Ten Item Personality Inventory, PSS-J = Japanese version of the Perceived Stress Scale.

Supplementary Table S4. Pearson and Spearman correlations (with BCa bootstrap 95% CIs) for key associations and rank-based partial correlations

	N	Pearson's *r* [95% CI]	Spearman's *ρ* [95% CI]
Age - Frequency of drinking alcoholic beverages (all contexts)	1279	.11 [.06, .17]	.08 [.03, .14]
Age - Frequency of listening to music	1275	−.19 [−.25, −.14]	−.19 [−.24, −.14]
Age - X	1275	.03 [−.03, .09]	.03 [−.03, .08]
Age - Y	1275	.02 [−.03, .07]	.04 [−.02, .09]
Age - Z	1275	−.13 [−.18, −.07]	−.13 [−.19, −.08]
X - Z (Rank-based partial correlation)	1275	.33 [.27, .38]	.35 [.30, .41]

Note. Pairs were selected based on primary hypotheses.

There were each four missing values for the data on the frequency of listening to music and tendency of doing each behavior when experiencing negative emotions.

X = tendency of drinking alcoholic beverages without listening to music when experiencing negative emotions,

Y = tendency of drinking alcoholic beverages with listening to music when experiencing negative emotions,

Z = tendency of listening to music without drinking alcoholic or caffeinated beverages when experiencing negative emotions.

Supplementary Table S5. Partial correlations between frequency of drinking alcoholic beverages and the DMQ-RJ subscale scores

	DMQ-RJ			
	Social	Coping	Enhancement	Conformity
**Frequency of drinking alcoholic beverages**				
All contexts	.18**	.26**	.33**	.11**
Alone context	.12**	.24**	.27**	.11**
Not-alone context (All − Alone)	.13**	.10**	.18**	.02

Note . Data analysis was limited to respondents who scored < 8 in the Alcohol Use Disorders Identification Test.

Each frequency was self-reported as the number of days (0-30) in which each behavior was performed within 30 days.

Control variables were gender and self-perceived ability to metabolize alcohol.

DMQ-RJ = Japanese version of the Drinking Motives Questionnaire-Revised.

N = 1279, ** $p < .01$

Supplementary Table S6. Partial correlations between frequency of listening to music and the B-MMR-J subscale scores

	B-MMR-J						
	Entertainment	Revival	Strong sensation	Diversion	Discharge	Mental work	Solace
**Frequency of listening to music**	.48**	.47**	.48**	.41**	.23**	.41**	.40**

Note . Data analysis was limited to respondents who scored < 8 in the Alcohol Use Disorders Identification Test.

Frequency was self-reported as the number of days (0-30) of listening to music within 30 days.

Control variables was gender.

B-MMR-J = Japanese version of the Brief Music in Mood Regulation Scale.

There were missing values for the data on the frequency of listening to music for three in male and one in female.

N = 1275, ** p < .01

Supplementary Table S7. Pearson's correlation coefficients between tendency of alcohol/music use when experiencing negative emotions, DMQ-R, and B-MMR

	N	**Tendency of doing each behavior when experiencing negative emotions**		
		Drinking alcoholic beverages without listening to music	Drinking alcoholic beverages with listening to music	Listening to music without drinking alcoholic/caffeinate d beverages
**DMQ-RJ (Z-score)**	926			
Social		−.14**	−.10**	−.10**
Coping		.21**	.12**	.19**
Enhancement		.25**	.16**	−.01
Conformity		−.31**	−.17**	−.07*
**B-MMR-J (Z-score)**	1151			
Entertainment		−.03	.01	−.11**
Revival		.09**	.08**	.16**
Strong Sensation		−.02	−.04	−.17**
Diversion		.01	−.03	.26**
Discharge		−.08**	−.04	−.25**
Mental Work		.01	−.00	.07*
Solace		.06*	.04	.22**

Note . Data analysis was limited to respondents who scored < 8 in the Alcohol Use Disorders Identificatic

Each tendency was self-reported on a five-point Likert scale (from 1 = *Never do it* to 5 = *Always do it*).

Abbreviations: DMQ-RJ = Japanese version of the Drinking Motives Questionnaire-Revised, B-MMR-J = Japanese version of the Brief Music in Mood Regulation Scale.

The Z-scores for DMQ-RJ could not be calculated for 353 participants.

The Z-scores for B-MMR-J could not be calculated for 128 participants.

** $p < .01$, * $p < .05$

Supplementary Table S8. Pearson correlation analysis for selecting mediator variables M1 and M2

		N	*M* (*SD*)	1	2	3	4	5	6	7	8	9	10	11	12	13	14	15	16	17
**1**	**Age**	1279	40.20 (9.94)	—																
	**TIPI-J**	1279																		
**2**	Extraversion		7.01 (2.93)	.15**	—															
**3**	Agreeableness		9.56 (2.50)	.10**	.05	—														
**4**	Conscientiousness		8.06 (2.98)	.18**	.27**	.42**	—													
**5**	Neuroticism		8.42 (2.80)	−.14**	−.30**	−.37**	−.41**	—												
**6**	Openness to Experience		7.12 (2.80)	.08**	.37**	.16**	.29**	−.28**	—											
	**CERQ-short-RJ**	1279																		
**7**	Self-blame		6.39 (1.90)	−.06*	−.10**	.03	−.04	.18**	−.12**	—										
**8**	Acceptance		6.75 (1.89)	.03	−.06*	.13**	.04	.05	−.02	.59**	—									
**9**	Rumination		6.47 (1.98)	−.06*	−.13**	−.03	−.03	.36**	−.05	.52**	.48**	—								
**10**	Positive refocusing		5.74 (2.00)	−.03	.11**	.16**	.08**	−.22**	.20**	.18**	.27**	.12**	—							
**11**	Refocus on planning		6.90 (1.89)	.14**	.09**	.26**	.26**	−.12**	.19**	.36**	.55**	.41**	.33**	—						
**12**	Positive reappraisal		5.99 (2.05)	.11**	.23**	.25**	.29**	−.27**	.33**	.30**	.47**	.22**	.50**	.60**	—					
**13**	Putting into perspective		5.72 (1.82)	.03	.07*	.11**	.06*	−.09**	.15**	.34**	.46**	.28**	.52**	.41**	.55**	—				
**14**	Catastrophizing		6.14 (1.96)	−.03	−.09**	−.01	.05	.25**	.04	.50**	.46**	.72**	.12**	.44**	.27**	.31**	—			
**15**	Other-blame		5.00 (1.84)	−.07*	−.02	−.11**	−.04	.02	.12**	−.07*	.04	.27**	.25**	.12**	.12**	.22**	.31**	—		
**16**	**PSS-J / Total score**	1279	20.21 (6.50)	−.08**	−.27**	−.32**	−.26**	.54**	−.21**	.17**	.10**	.38**	−.20**	−.04	−.22**	−.07*	.33**	.10**	—	
**17**	**Diversion of B-MMR-J (Z-score)**	1151	−0.19 (1.50)	−.09**	−.13**	−.09**	−.07*	.15**	−.03	.06	−.02	.07*	.02	−.06	−.03	.02	.06	.01	.16**	—

Note . Data analysis was limited to respondents who scored < 8 in the Alcohol Use Disorders Identification Test.

TIPI-J = Japanese version of the Ten Item Personality Inventory.

CERQ-short-RJ = Revised Japanese version of the Cognitive Emotion Regulation Questionnaire.

PSS-J = Japanese version of the Perceived Stress Scale

B-MMR-J = Japanese version of the Brief Music in Mood Regulation Scale.

The Z-scores for B-MMR-J could not be calculated for 128 participants.

** $p < .01$, * $p < .05$

Supplementary Table S9. Partial correlations between tendency of doing each behavior when experiencing negative emotions and the CERQ-short-RJ subscale scores

	**CERQ-short-RJ**								
	Self-blame	Acceptance	Rumination	Positive refocusing	Refocus on planning	Positive reappraisal	Putting into perspective	Catastrophizing	Other-blame
**Tendency of doing each behavior when experiencing negative emotions**									
Drinking alcoholic beverages without listening to music	.02	.00	−.00	.04	−.03	−.01	.02	.05	.07*
Drinking alcoholic beverages with listening to music	−.00	−.05	−.00	.03	−.08**	.10**	.06*	.06*	.06*
Listening to music without drinking alcoholic/caffeinated beverages	.03	−.01	.05	.09**	−.04	.08**	.03	.04	−.02

Note . Data analysis was limited to respondents who scored < 8 in the Alcohol Use Disorders Identification Test.

Each tendency was self-reported on a five-point Likert scale (from 1 = *Never do it* to 5 = *Always do it*).

Control variables were gender, self-perceived ability to metabolize alcohol, and the remaining 8 cognitive emotion regulation strategies other than the relevant strategy.

There were missing values for three in male and one in female.

CERQ-short-RJ = Revised Japanese version of the Cognitive Emotion Regulation Questionnaire.

N = 1275, ** p < .01, * p < .05

Supplementary Figure S1. Representative Q–Q plots for assumption checks

(a) **(b)** **(c)**

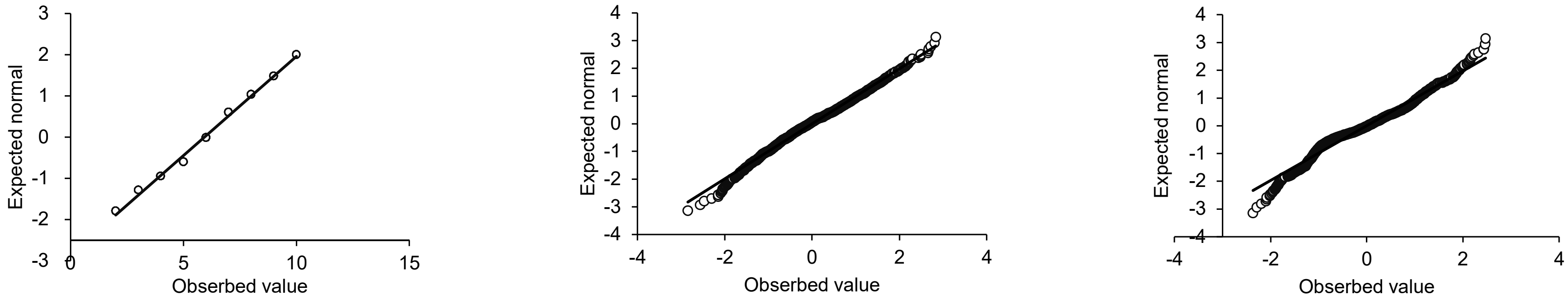

Note . (a) Normal Q–Q plots for the Diversion subscale score of the CERQ-short-RJ by gender (Welch's *t* -test). Both groups were inspected; representative plots for the male group are shown.

(b) Q–Q plot of standardized residuals for the correlation model (X = age, Y = Diversion subscale score of the B-MMR-J Z-score).

(c) Q–Q plot of residuals from a rank-based partial correlation model (Spearman-type), where residuals were obtained by regressing the rank-transformed X and Y on the covariates

(X = tendency of drinking alcoholic beverages without listening to music when experiencing negative emotions; Y = tendency of listening to music without drinking alcoholic or caffeinated beverages when experiencing negative emotions; the covariates = age, gender [dummy-coded], and self-perceived ability to metabolize alcohol.)

Supplementary Figure S2. Mean frequency (all contexts) of drinking alcoholic beverages among each age

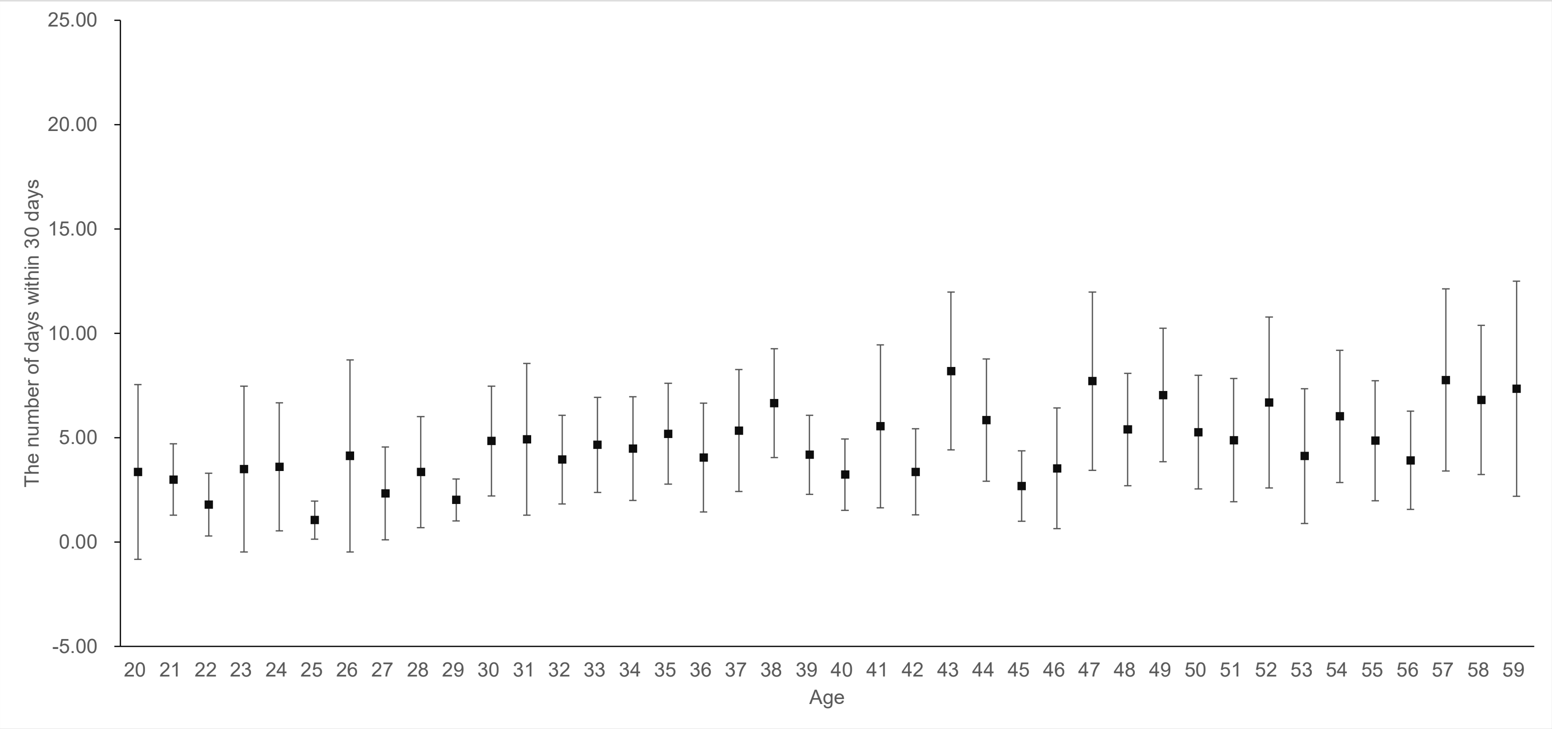

Note . Data analysis was limited to respondents who scored < 8 in the Alcohol Use Disorders Identification Test.
Error bars represent 95% CI.

Supplementary Figure S3. Mean frequency of listening to music among each age

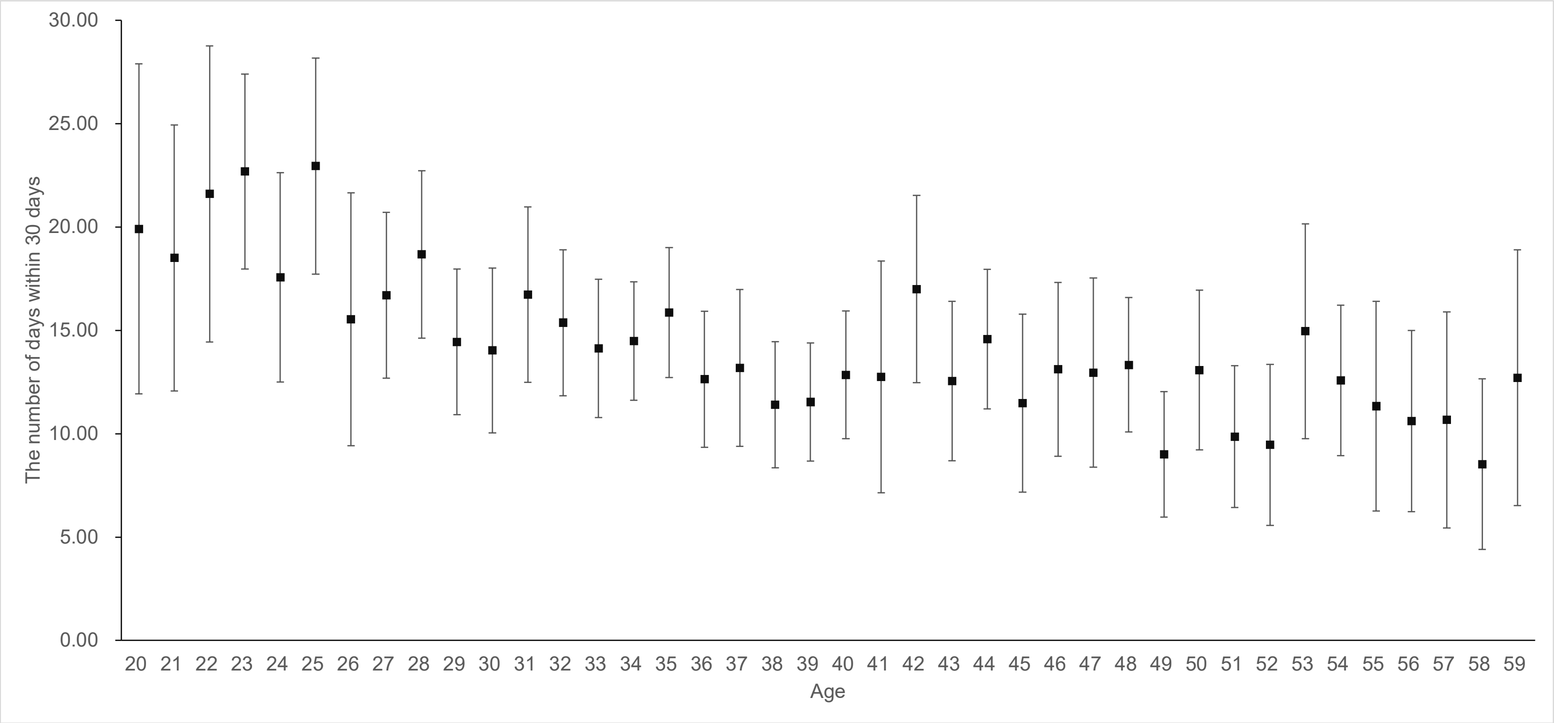

Note . Data analysis was limited to respondents who scored < 8 in the Alcohol Use Disorders Identification Test.
Error bars represent 95% CI.
